# A Hybrid Technique for the Periodicity Characterization of Genomic Sequence Data

**DOI:** 10.1155/2009/924601

**Published:** 2009-03-02

**Authors:** Julien Epps

**Affiliations:** 1 School of Electrical Engineering and Telecommunications, The University of New South Wales, Sydney NSW 2052, Australia; 2 National Information Communication Technology Australia (NICTA), Australian Technology Park, Eveleigh 1430, Australia

## Abstract

Many studies of biological sequence data have examined sequence structure in terms of periodicity, and various methods for measuring periodicity have been suggested for this purpose. This paper compares two such methods, autocorrelation and the Fourier transform, using synthetic periodic sequences, and explains the differences in periodicity estimates produced by each. A hybrid autocorrelationâ€”integer period discrete Fourier transform is proposed that combines the advantages of both techniques. Collectively, this representation and a recently proposed variant on the discrete Fourier transform offer alternatives to the widely used autocorrelation for the periodicity characterization of sequence data. Finally, these methods are compared for various tetramers of interest in *C. elegans* chromosome I.

## 1. Introduction

The detection of structure within the DNA sequence has long captivated the interest of the research community. Among the various statistical characterizations of sequence data, one measure of structure within sequences is the degree of correlation or periodicity at various displacements along the sequence. Periodicity characterization of sequence data provides a compact and informative representation that has been used in many studies of structure within genomic sequences, including DNA sequence analysis [1], gene and exon detection [2], tandem repeat detection [3], and DNA sequence search and retrieval [4].

To measure such periodicity, autocorrelation has been widely employed [1, 5–11]. Similarly, Fourier analysis and its variants have been used for periodicity characterization of sequences [4, 9, 12–24]. In some cases [25, 26], the Fourier transform of the autocorrelation sequence has also been computed, however using existing symbolic-numeric mappings such as binary indicator sequences [27], this transform can also be calculated without first determining the autocorrelation. Other recent promising approaches to periodicity characterization for biological sequences include the periodicity transform [28], the exactly periodic subspace decomposition [3], and maximum-likelihood statistical periodicity [29], however these techniques have yet to be adopted by biologists for the purposes of sequence structure characterization.

Studies of structure within sequences, such as those referenced above, have tended to use either the autocorrelation or the Fourier transform, and to the author's knowledge, the limitations of each have not been compared in this context. In this paper, the limitations of both approaches are investigated using synthetic symbolic sequences, and caveats to their characterization of sequence data are discussed. A hybrid approach to periodicity characterization of symbolic sequence data is introduced, and its use is illustrated in a comparative manner on a study of tetramers in *C. elegans*.

## 2. Periodicity Measures for Symbolic Sequence Characterization

### 2.1. Definition of Periodicity

Perhaps the most common definition of exact periodicity in a general sequence  is (1)

for some . Assuming  can be represented numerically as , this definition admits the following decomposition: (2)

where (3)

is the numerical representation of a repeated symbol or pattern, and  is a periodic binary impulse train: (4)

While this expression of  in terms of a binary impulse train is perhaps not so common in signal processing of numerical sequences, the reverse is true for DNA sequences, which have been represented numerically using binary indicator sequences [27] in many studies (e.g., [13, 19, 23, 24, 30]).

### 2.2. Autocorrelation

The autocorrelation of a finite length numerical sequence  is defined as (5)

where *n* is the sequence index, *ρ* is the lag, and *N* is the length of the sequence. The application of the autocorrelation as defined in (5) to a symbolic sequence  requires a numerical representation . The binary indicator sequences [27], which are sufficiently general as to form the basis for many different representations of DNA sequences, are employed in this analysis to represent  in terms of *M* binary signals: (6)

where *M* is the number of symbols (or patterns of symbols, such as a polynucleotide) , to which the numerical values  are assigned, respectively, resulting in *M* components . Assuming , the numerical representation can thus be unambiguously expressed as (7)

Note that applying the decomposition in (2) to an exactly periodic sequence results in  comprising a sequence of the numerical values  that correspond to the repeated pattern of symbols.

Alternatively, the autocorrelation can be defined directly on a symbolic sequence , as used in [20]: (8)

so that the autocorrelation at a lag, or period,  for a symbol (or pattern of symbols) is simply the count of the number of instances of that symbol at a spacing of *ρ*.

**Figure 1:**
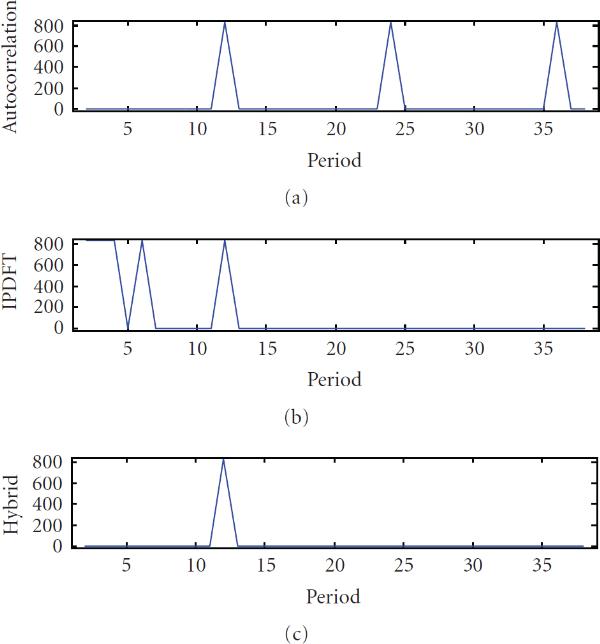
**Periodicity characterization of the period-12 synthetic signal  using (a) autocorrelation, (b) integer period DFT, and (c) hybrid autocorrelation-IPDFT**.

Consider now a sequence containing a symbol (or pattern of symbols)  that repeats with exactly period *p*, so that the numerical representation of the sequence has a component . The autocorrelation of this component , for a segment of finite length *N*, has the following expression: (9)

where  is the energy of  over a segment of finite length *N*. Thus a shortcoming of the autocorrelation for sequence characterization is that an exactly *p*-periodic sequence will show not only a peak at , but also peaks at values of  that are integer multiples of *p* (an example is given in Figure 1(a)). Note that similar artifacts can be found in other periodicity detection methods (e.g., [29]).

### 2.3. Fourier Interpretation of Periodicity

In many applications, including sequence analysis, the discrete Fourier transform has been used to determine the periodic component(s) of a numerical sequence . The discrete Fourier transform (DFT) of a numerical sequence  is defined as (10)

where *k* is the discrete frequency index. Since the DFT has sinusoidal basis functions, the notion of periodicity in the Fourier sense is described in terms of the frequencies of those basis functions onto which the projections of  are the largest in magnitude. That is, the magnitude of the DFT at a frequency *k*, , is often taken as an estimate of the relative amount of that frequency component occurring in  [13, 19, 23, 24], from which the relative contribution of a particular period  can be estimated.

Assuming a numerical representation  of the kind shown in (7), the linearity property of the DFT means that the DFT of a symbolic sequence  can be determined as (11)

where the  are determined according to (10).

For the purposes of characterizing sequence data using periodicity, it can be noted that positive integer periods are generally of most interest. This means firstly that *N* and *k* need to be carefully chosen to allow fast Fourier transform-based calculation of  for periods , where *P* is the longest period to be estimated. Secondly, calculating the DFT at other frequencies  is unnecessary. For these reasons, the integer period DFT (IPDFT) was proposed as an alternative to the DFT [19]: (12)

Using a similar process to that described above in (10) and (11), the numerical representation of a symbolic sequence  can also be transformed using the IPDFT to produce a spectrum  that is linear in period (*ρ*) rather than in frequency (*k*). For the periodicity characterization of sequences, usually the magnitude  is of greatest interest. Some care is needed in the interpretation of the IPDFT, since for a binary periodic sequence such as  of fixed length *N*,  will decrease for longer periods due to the fact that the energy of  is .

Consider now the effect of representing an exactly periodic sequence component  using the IPDFT. From (2) and the convolution theorem, , where  is the IPDFT of . In particular, if  is assumed to be aperiodic, consider the IPDFT of : (13)

where . That is,  is relatively large for , and relatively small for . From this, we see that a shortcoming of Fourier transform approaches such as the IPDFT for sequence characterization by periodicity is that they produce not only a peak at , but also peaks at values of  that are integer divisors of the period *p* (see example in Figure 1(b)). For the DFT, this effect is also seen, but instead for indices whose value is  (i.e., harmonics of the frequency  with integer frequency indices).

### 2.4. Periodicity of a Synthetic Sequence Using Autocorrelation and DFT

To illustrate the shortcomings of the autocorrelation and DFT discussed in Sections 2.2 and 2.3, consider the periodicity characterization of an example signal  (i.e., exact monomer periodicity ), where  and . The autocorrelation and IPDFT are shown in Figures 1(a) and 1(b), respectively, from which the ambiguities in period estimate discussed in Sections 2.2 and 2.3 can be clearly seen.

## 3. Hybrid Autocorrelation-IPDFT Periodicity Estimation

### 3.1. Hybrid Autocorrelation-IPDFT

From Figure 1, it is apparent that the autocorrelation and IPDFT are complementary, and that their combination can improve periodicity estimation. This is the motivation for the hybrid autocorrelation-IPDFT period estimate: (14)

For the simple example signal  from Section 2.4, the calculation of  results in a single, unambiguous periodicity estimate, as seen in Figure 1(c).

An alternative, more flexible formulation is (15)

where , which may be helpful for biologists who have conventionally used either the autocorrelation () or the Fourier transform (). For the purpose of sequence periodicity visualization, for example,  could be represented as a parameter available for real-time control, so that a biologist viewing a periodicity characterization of a sequence might subjectively assign a relative weight to each of the autocorrelation and Fourier transform components. Care is needed, however, with the application of (15), since  is only well defined for  for all . Note that this is satisfied by the autocorrelation defined in (8), in addition to a number of DNA numerical representations (several example representations are discussed in [30]). It is further noted that (14) and (15) do not have a straightforward physical interpretation, in contrast to  and .

**Figure 2:**
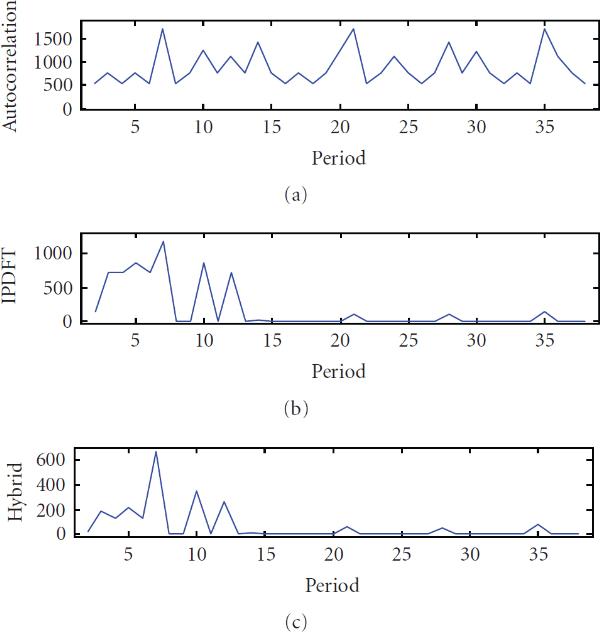
**Periodicity characterization of a period-7, 10 and 12 synthetic signal using (a) autocorrelation, (b) integer period DFT, and (c) hybrid autocorrelation-IPDFT**.

Applying the hybrid autocorrelation-IPDFT period estimate to another example, synthetic signal with multiple exact periodic components () further illustrates the shortcomings of the autocorrelation and IPDFT, and suggests the hybrid approach as suitable for periodicity analyses, as seen in Figure 2.

### 3.2. Evaluation of Periodicity Estimation in Noise

In the absence of an obvious objective evaluation metric for periodicity characterization approaches, one limited approach is to compare their accuracies for the problem of estimating a single periodic component that has been obscured by noise. Specifically, suppose a periodic binary impulse train  is degraded by random binary noise, simulating the effect of the DNA substitution process, to produce a binary pseudo-periodic signal . Then estimates of the signal periodicity using each of the autocorrelation, integer period DFT and hybrid autocorrelation-IPDFT can be calculated, respectively, as (16)

where  is calculated using (14) throughout both this section and Section 4.

**Figure 3:**
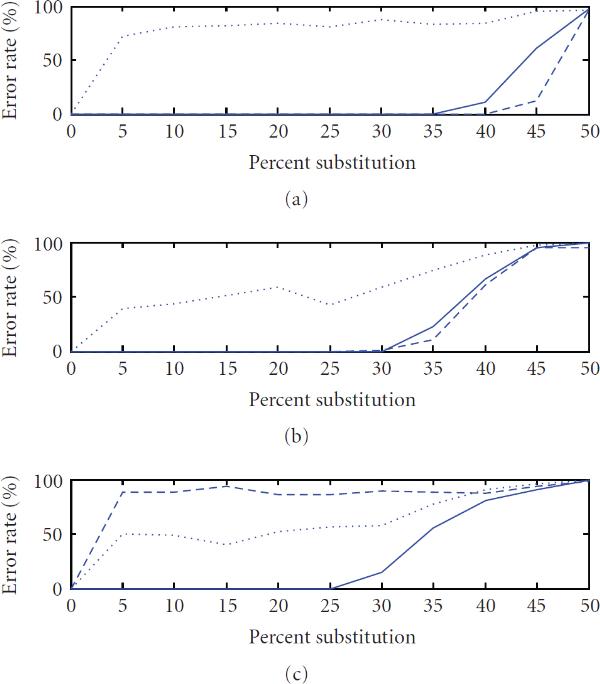
**Error rate versus substitutions averaged over 100 instances of sequences of length 10000 with (a) , (b) , (c) , for period estimates using autocorrelation (), integer period DFT (), and hybrid autocorrelation-IPDFT (—)**.

**Figure 4:**
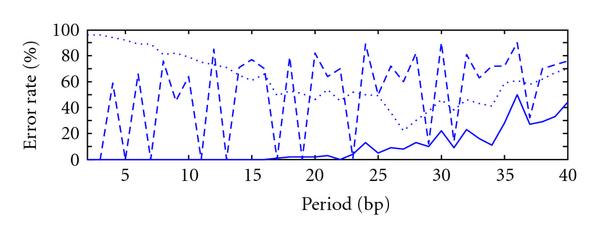
**Error rate versus period averaged over 100 instances of sequences of length 10000 with a substitution rate of 30%, for period estimates using autocorrelation (), integer period DFT (), and hybrid autocorrelation-IPDFT (—)**.

A comparison of the periodicity estimates was conducted by generating synthetic periodic signals of length , introducing various amounts of substitution (noise) and estimating , , and . This process was repeated 100 times for each combination of period and substitution rate tested. The resulting average period error rates are shown as a function of substitution rate for three example values of period *p* in Figure 3 (*p* small, *p* larger and prime, and *p* larger and highly composite), and as a function of the period in Figure 4. These results confirm earlier observations that the IPDFT provides more robust period estimates for prime periods than the autocorrelation, while the reverse is true for highly composite periods. The results also show that the hybrid technique is often able to provide a lower period error rate than either the autocorrelation or the IPDFT. Exceptions to this occur for some prime periods (see Figure 4), where the poorer performance of the autocorrelation seems to slightly adversely affect the hybrid estimate  relative to the IPDFT-only estimate .

### 3.3. Evaluation of Multiple Periodicity Estimation

**Figure 5:**
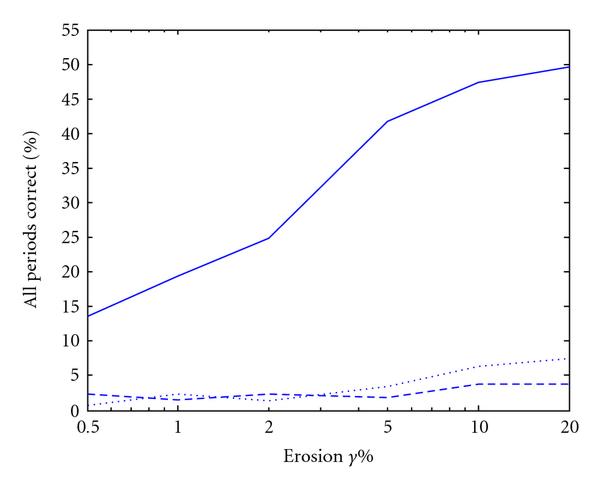
**Percentage of sequence instances for which all three periods were correctly estimated in order of strength versus erosion , over 500 instances of sequences of length 10000 with three randomly chosen integer periodic components, estimated using autocorrelation (), integer period DFT (), and hybrid autocorrelation-IPDFT (—)**.

For periodicity characterization, a more relevant evaluation criterion is the extent to which all periodicities present can be detected correctly. Since an exhaustive evaluation is impractical, in this work, synthetic sequences comprising three randomly chosen integer periodic components  were constructed, and the frequency with which all three periods were correctly detected was measured. When multiple perfectly periodic components are present in a binary signal, the shorter periods will be favoured during estimation, as a result of their greater occurrence in a fixed-length signal. Hence, when combining three periodic components, the shorter period components were randomly eroded to give an equal occurrence between all periods. In the general case of multiple periodicities, some periodic components will be stronger than others. To simulate this, the -periodic component was further randomly eroded by  % and the -periodic component was further randomly eroded by  %, that is, larger values of  correspond to a more dominant  component. Erosions of greater than about 20% were experimentally found to degrade the accuracy of all three period estimates, using all methods. Finally, the percentage of instances for which the periods , , and  were correctly estimated in correct order of strength according to the 3-best period estimates, calculated similarly to equations (16), was determined. The results, shown in Figure 5, strongly support the validity of the proposed hybrid autocorrelation-IPDFT technique relative to the autocorrelation and IPDFT.

It is noted that the signal processing literature includes examples of methods for detecting multiple periodic signal components, such as the MUSIC algorithm [31]. For comparative purposes, the above experiment was repeated employing MUSIC to estimate the strengths of the periodic components. Results indicated that MUSIC was unable to consistently estimate either the periods or the relative strengths of the three components, returning no instances of all three periods correct and in the correct order. The dominant period estimate often contained the common factors of two or more of the true periodic components, an artifact attributable to the superposition of harmonic spectra reinforcing multiples of the individual component fundamentals that coincide in frequency. Two assumptions of MUSIC are not valid for this application: (i) the periodic components are not sinusoidal (although they can be represented as a harmonic series of sinusoids), (ii) the periodic components and noise may not be uncorrelated.

## 4. Application to DNA Sequence Data

Having discussed the differences between the autocorrelation and DFT for synthetic sequences, we now investigate the effect of using the IPDFT and hybrid autocorrelation-IPDFT in place of the autocorrelation on real sequence data. Numerous researchers have used autocorrelation [1, 5–10, 32]; here we compare with examples from the study of tetramer periodicity in the *C. elegans* genome using autocorrelation by Kumar et al. [1].

**Figure 6:**
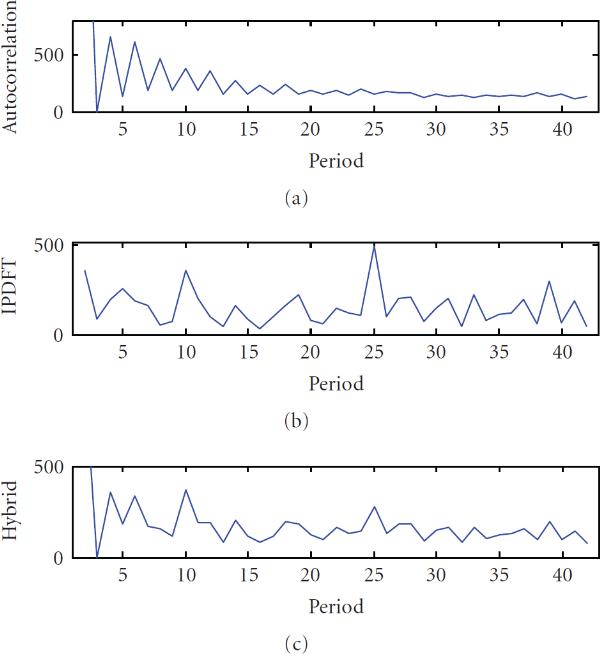
**(a) Autocorrelation from **[1], **(b) integer period DFT magnitude, and (c) hybrid autocorrelation-IPDFT of TATA tetramers from *C. elegans* chromosome I**.

In the investigation of TATA tetramers, particular mention was made of the strong period-2 component [1], which features prominently in estimates by all three techniques, as seen in Figure 2. In the autocorrelation estimate (Figure 6(a)), the period-10 component appears to have been virtually completely masked by the period-2 component. In contrast, the period-10 component features strongly in the IPDFT (Figure 6(b)) and hybrid (Figure 6(c)) estimates. Although this period-10 component was not mentioned in the analysis of TATA tetramers specifically, it was found to be characteristic of all other *C. elegans* tetramers analyzed in [1].

Note also that the IPDFT reveals a strong period-25 component, not at all evident in the autocorrelation. This surprising result was verified by constructing a synthetic sequence with perfect periodic components at  and , and examining its autocorrelation and IPDFT. The autocorrelation of the sequence did not display visually any significant peak at  until the period-2 component had been eroded by at least 80%. In contrast, the IPDFT showed a clear peak at  with no period-2 erosion at all. The period-25 component has rarely been noted in previous literature, however in [11], a filtered distribution of distances between TA dinucleotides shows a strong peak at , which Salih et al. attribute to a 5-base periodicity associated with the period-10 consensus sequence structure for *C. elegans*.

**Figure 7:**
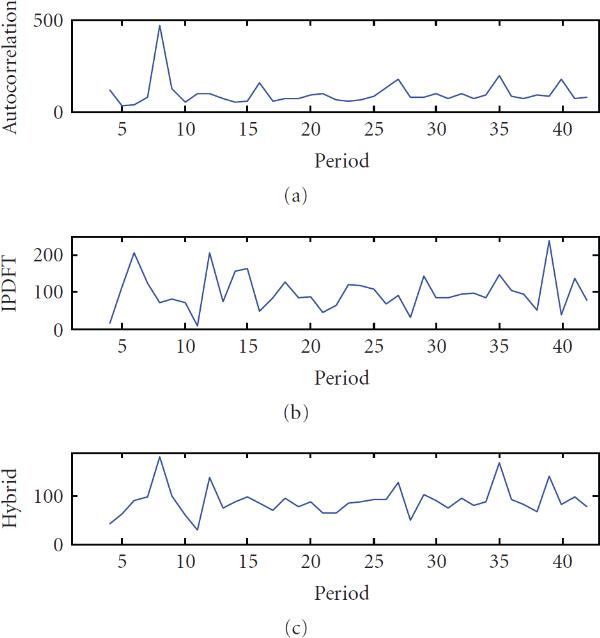
**(a) Autocorrelation from **[1]**, (b) integer period DFT magnitude, and (c) hybrid autocorrelation-IPDFT of TGCC tetramers from *C. elegans* chromosome I**.

In the investigation of TGCC tetramers (see Figure 7), the periodic components at 8 and 35 bp were noted in [1]. The proposed hybrid technique also produces peaks at these periods (mainly due to the autocorrelation in this instance), however it additionally finds period-12 and period-39 components. Note that the IPDFT produces a strong peak at a 6 bp period (presumably due to being an integer divisor of 12), however in the hybrid result, this is effectively suppressed by the autocorrelation.

**Figure 8:**
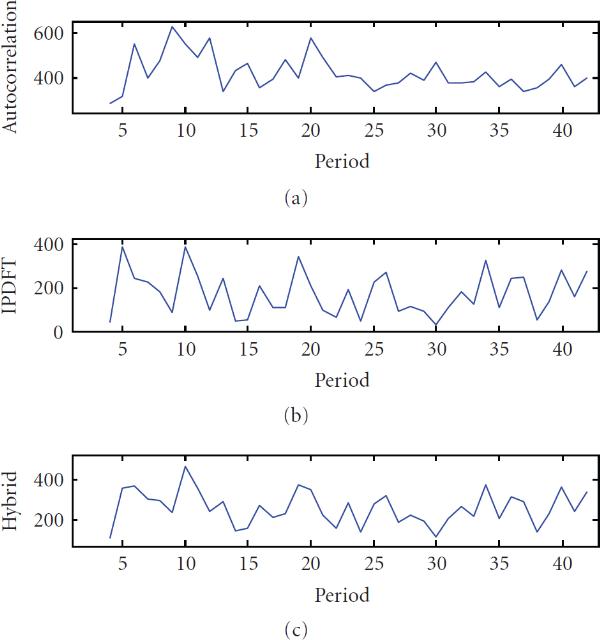
**(a) Autocorrelation from **[1]**, (b) integer period DFT magnitude, and (c) hybrid autocorrelation-IPDFT of AGAA tetramers from *C. elegans* chromosome I**.

In [1], mention is made of the period-10 and 11 behaviour of AGAA tetramers. As seen in Figure 8, the autocorrelation finds a dominant peak at 9 bp, while the hybrid technique is more convincing in revealing period-10 behavior. Note that, as previously, the period-5 IPDFT component (presumably due to the 10 bp periodicity) is effectively attenuated in the hybrid result.

**Figure 9:**
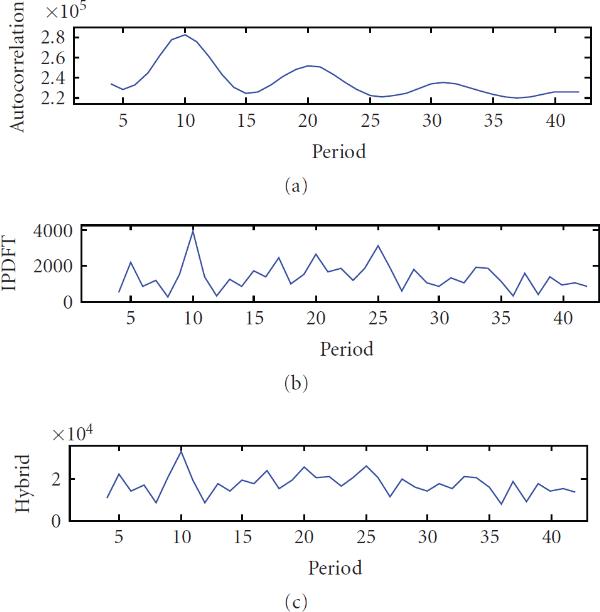
**(a) Autocorrelation from **[1]**, (b) integer period DFT magnitude, and (c) hybrid autocorrelation-IPDFT of WWWW tetramers from *C. elegans* chromosome I**.

In the investigation of WWWW tetramers (where W represents either A or T), the autocorrelation (Figure 9(a)), as in [1], is dominated by the period-10 component. A very similar characteristic is observed in the distribution of distances between TT to TT dinucleotides in [11], and in the distribution of AAAA to AAAA tetramer distances in [33], suggesting a strong influence by these motifs. While the dominance of the period-10 component is similar for the IPDFT, it also detects a relatively strong period-25 component, perhaps due to TA dinucleotide periodicity, as discussed above for TATA tetramers. In this example, the hybrid autocorrelation-IPDFT result is biased towards the IPDFT, as a result of the IPDFT having a larger dynamic range than the autocorrelation. Here, the effect is not detrimental, having the effect of suppressing the spurious peaks at periods 20, 30, and 40, however in other applications it may be desirable to offset the autocorrelation and/or IPDFT to produce a minimum value of zero prior to calculating the hybrid autocorrelation-IPDFT period estimate.

## 5. Conclusion

This paper has made two contributions to the periodicity characterization of sequence data. Firstly, the origins of ambiguities in period estimates for symbolic sequences due to multiples or sub multiples of the true period in the autocorrelation and Fourier transform methods, respectively, were explained. This is significant because these two methods account for perhaps the majority of the periodicity analysis seen in biology literature, and yet, to the author's knowledge, their limitations have not been discussed in this context. Secondly, a hybrid autocorrelation-IPDFT technique for periodicity characterization of sequences has been proposed. This technique has been shown to provide improved accuracy relative to the autocorrelation and IPDFT for period estimation in noise and multiple periodicity estimation, for synthetic sequence data. Comparative results from a preliminary investigation of tetramers in *C. elegans* chromosome I suggest that the proposed approach yields estimates that are consistently less prone to attribute significance to integer multiples or divisors of the true period(s). Thus, the hybrid autocorrelation-IPDFT is putatively advanced as a useful tool for biologists in their quest to reveal and explain structure within biological sequences. Future work will include studies of different types of periodicity in sequence data from other organisms, using IPDFT-based and hybrid techniques.
